# A Prospective Study Comparing the Diagnostic Ability of Abdominal Ultrasonography with Computed Tomography in Cases Suspected of Retaining the Patency Capsule

**DOI:** 10.1002/deo2.70331

**Published:** 2026-04-19

**Authors:** Tomoharu Matsuura, Satoshi Osawa, Moriya Iwaizumi, Ryota Inoue, Wataru Inui, Tomoyuki Niwa, Kenichi Takahashi, Takatoshi Egami, Keisuke Inagaki, Tomohiro Takebe, Tatsuhiro Ito, Satoru Takahashi, Shunya Onoue, Yusuke Asai, Kiichi Sugiura, Natsuki Ishida, Mihoko Yamade, Yasushi Hamaya, Takanori Yamada, Ken Sugimoto

**Affiliations:** ^1^ Department of Laboratory Medicine Hamamatsu University School of Medicine Shizuoka Japan; ^2^ Department of Advanced Medical Science for Regional Collaboration Hamamatsu University School of Medicine Shizuoka Japan; ^3^ Department of Endoscopic and Photodynamic Medicine Hamamatsu University School of Medicine Shizuoka Japan; ^4^ First Department of Medicine Hamamatsu University School of Medicine Shizuoka Japan

**Keywords:** abdominal ultrasonography, capsule retention, Crohn's disease, patency capsule, small bowel capsule endoscopy

## Abstract

**Objectives:**

Patency capsule (PC) testing is widely used to assess small‐bowel patency before small‐bowel capsule endoscopy (SBCE). Although abdominal computed tomography (CT) reliably identifies PC location, routine use is limited by radiation exposure and cost. Abdominal ultrasonography (AUS) is a noninvasive, radiation‐free alternative; however, its diagnostic accuracy for PC localization remains unclear.

**Methods:**

We conducted a prospective, exploratory, single‐center study in patients in whom PC excretion could not be confirmed within the predefined observation period. All patients underwent AUS followed by CT on the same day. The primary endpoint was the sensitivity of AUS for detecting small‐bowel PC retention using CT as the reference standard. Secondary endpoints included the PC visualization rate, diagnostic accuracy for small bowel versus colon classification, six‐segment agreement between AUS and CT, and SBCE retention after AUS‐based assessment.

**Results:**

Thirty‐four patients were included. AUS visualized the PC in 28 patients (82.4%). All six patients with small‐bowel retention on CT were correctly identified as “small bowel” by AUS (sensitivity 100%; 95% confidence interval [CI], 61.0–100). Among 28 patients with colonic PC on CT, AUS classified 22 as “colon,” yielding a sensitivity of 78.6% (95% CI, 60.5–89.9). Six‐segment agreement between AUS and CT was 82.4% overall and 100% among visualized cases. No SBCE retention occurred after AUS‐based colonic classification.

**Conclusions:**

AUS accurately detected small‐bowel PC retention and showed good agreement with CT when visualization was achieved. Although non‐visualized findings require cautious interpretation, AUS may serve as a useful first‐line imaging modality for evaluating PC location prior to SBCE.

**Trial Registration:**

N/A.

## Graphical abstract

This prospective study evaluates the diagnostic performance of abdominal ultrasonography (AUS) for localizing patency capsules using CT as the reference standard. AUS accurately detects small‐bowel retention and demonstrates high agreement with CT when visualization is achieved. AUS may serve as a reliable first‐line, radiation‐free modality for triaging patients prior to small‐bowel capsule endoscopy.

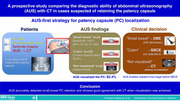

## 1 Introduction

Patency capsule (PC) testing is widely used to assess small‐bowel patency prior to small‐bowel capsule endoscopy (SBCE), particularly in patients with known or suspected strictures [1, 2, 3, 4]. The accurate determination of PC location is essential for ensuring the safe performance of SBCE [5, 6], as unrecognized small‐bowel retention can result in capsule impaction, the need for endoscopic or surgical retrieval, and delays in diagnosis [7, 8]. Abdominal computed tomography (CT) is considered a reliable modality for confirming PC location; however, the routine use of CT is limited by radiation exposure, cost, and availability [4, 9]. Therefore, a simple, noninvasive, and radiation‐free alternative method for evaluating PC location is highly desirable in clinical practice.

Abdominal ultrasonography (AUS) is widely available, inexpensive, and does not involve ionizing radiation, making it an attractive modality for evaluating the gastrointestinal tract [10]. Several reports have suggested that AUS may be capable of identifying the PC within the intestines [11, 12, 13]. However, most previous studies have been retrospective or descriptive in nature, have not used CT as a strict reference standard, or have focused on visualization feasibility rather than diagnostic accuracy. Consequently, the clinical utility of AUS for reliably determining PC location—particularly in distinguishing small‐bowel retention from colonic passage—remains insufficiently established.

To address this gap, we conducted a prospective, single‐center diagnostic accuracy study to evaluate the performance of AUS in identifying PC location, using CT as the reference standard. Our primary objective was to determine the ability of AUS to detect small‐bowel PC retention. Secondary objectives included assessing the overall visualization rate of AUS, segment‐specific agreement between AUS and CT, diagnostic accuracy for small bowel versus colon classification, and the clinical safety of proceeding to SBCE when AUS indicated colonic PC passage.

## 2 Methods

### 2.1 Study Design and Setting

This prospective, single‐center exploratory diagnostic‐accuracy study was conducted to evaluate the ability of AUS to identify the location of the PC using abdominal CT as the reference standard. In accordance with the Ethical Guidelines for Medical and Health Research Involving Human Subjects (Ministry of Education, Culture, Sports, Science and Technology and Ministry of Health, Labor and Welfare, Japan), study information, including the objectives, was disclosed on our hospital website with an opt‐out approach. The Ethics Committee of Hamamatsu University School of Medicine in Japan reviewed and approved the study protocol (institutional review board approval number: 21‐245).

### 2.2 Participants

This study was conducted on 157 consecutive cases who underwent PC testing at our institution between September 2022 and October 2025 for assessment of small‐bowel patency prior to SBCE. No formal a priori sample size calculation was performed, as this study was designed as an exploratory investigation. The inclusion criteria were: 1) cases in which PC had not been confirmed to have been excreted 30 h after swallowing PC and where residual PC was confirmed in the body by X‐ray, 2) patients who consented to this clinical study and provided written consent, and 3) patients who were able to undergo both an AUS and an abdominal CT scan immediately afterward on the day of PC assessment. Exclusion criteria were: 1) cases in which consent was withdrawn, and 2) cases in which the image information and written findings from the AUS and CT scan were insufficient for analysis.

### 2.3 Patency Capsule Protocol

**FIGURE 1:**
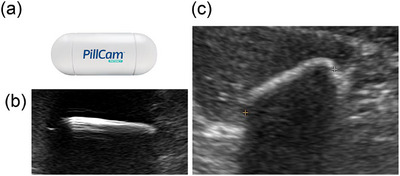
Ultrasound image of a patency capsule. (a) Patency capsule (PillCam Patency Capsule). (b) Ultrasound image of a patency capsule in water. (c) Ultrasound image of a patency capsule in the colon.

We used the tag‐less PillCam PC (Figure 1a; Covidien Japan, Medtronic, Japan), which has the same size and components as the conventional Agile PC with a radiofrequency identification tag [4, 14]. After 12 h of fasting, patients swallowed the PC with water at 9 AM. After 2 h, drinking water was allowed, and in the next 2 h, a meal was provided. Excretion of the PC was confirmed visually using a PC recovery kit during bowel movement until their outpatient visit the following evening. Small‐bowel patency was confirmed with a PC 30 h after its ingestion. ‘Confirmed patency’ was defined as the visual verification of an intact capsule excreted within 30 h. ‘Estimated patency’ was defined as the absence of PC evidence in the body via X‐ray at 30 h, according to our previous study [15]. If an X‐ray showed that PC remained inside the body, AUS was performed to record its location, and then a plain CT scan was performed immediately afterward to confirm its location.

### 2.4 Abdominal Ultrasonography

The AUS operator had access to past X‐ray results, and the estimated location of the PC was communicated by a gastroenterologist before the examination. CT findings were blinded until the AUS findings were fully documented. AUS was performed using the Aplio i700 system (Canon Medical, Tokyo, Japan) and the Aplio i800 system (Canon Medical, Tokyo, Japan). AUS was performed using a convex probe (PVU‐375BT or PVI‐475BX) with a frequency of 2.5 to 5 MHz and a linear probe (PLT‐704SBT or PLI‐605BX) with a frequency of 7–9 MHz. Patients had eaten an unlimited amount of food 3–4 h before the ultrasound examination (no bowel preparation was performed, and no dietary restrictions were instructed). The ultrasound examinations were performed by one board‐certified ultrasound specialist and one registered gastrointestinal sonographer, with 9 and 15 years of ultrasound experience, respectively. The AUS examination was limited to approximately 15 min to fit the routine clinical workflow and allow same‐day CT.

Observation began with the sigmoid colon around the bladder. If no PC was identified after observing the entire colon, examination of the small intestine was initiated, and observation of the small intestine was performed using the “lawn‐mower” technique [10]. The distinction between colon and small intestine was made based on a comprehensive assessment of intestinal peristalsis, the presence of intestinal mucosal folds, and continuity from the sigmoid colon. If a PC was identified, its course was recorded as the “rectum,” “sigmoid colon,” “descending colon,” “transverse colon,” “ascending colon,” or “small intestine.” Because PCs tend to migrate easily in the cecum, they were grouped under the ascending colon. The time required to identify a PC was calculated as the difference between the “time the PC was identified” and the “time the bladder image was taken.” If a PC could not be identified after 15 min or more of intestinal examination, the ultrasound examination was terminated, and a plain CT scan was performed, which was categorized as “ not visualized.”

### 2.5 CT (Reference Standard)

Plain (non–contrast‐enhanced) CT was performed immediately after AUS using standard abdominal imaging protocols [9]. The AUS‐to‐CT interval was defined as the time from completion of ultrasound acquisition to the start of CT; patients were instructed not to defecate during this interval and walked between the two suites. In all cases, CT was performed within 30 min after AUS. At least two board‐certified radiologists, each blinded to the AUS findings, independently assessed the CT images. Discrepancies were resolved by consensus. PC location was categorized into the same six anatomical segments used for AUS.

### 2.6 Endpoints

The primary endpoint was the sensitivity of AUS for detecting PC retention in the small bowel, using CT as the reference standard. AUS findings were classified as positive when the PC was identified in the small bowel. Secondary endpoints included the overall PC visualization rate on AUS, diagnostic accuracy for small bowel versus colon classification, segment‐level agreement between AUS and CT across six anatomical segments, and the occurrence of capsule retention during subsequent SBCE in patients judged to have colonic PC passage on AUS. Because non‐visualized findings may still represent small‐bowel retention, performance was also evaluated using three AUS classifications (“small bowel,” “colon,” and “not visualized”).

### 2.7 Statistical Analysis

Diagnostic accuracy of AUS for detecting small‐bowel PC retention was evaluated using CT as the reference standard. For the primary analysis, AUS findings were dichotomized as positive (PC identified in the small bowel) or negative (PC identified in the colon or not visualized); this operational definition did not exclude small‐bowel retention in non‐visualized cases. Sensitivity, specificity, positive and negative predictive values, and accuracy were calculated with 95% confidence intervals. Category‐specific performance for the three AUS classifications (“small bowel,” “colon,” and “not visualized”) was further assessed by calculating category‐specific sensitivities and predictive values. Six‐segment agreement between AUS and CT was calculated in all patients and in a per‐protocol subset with successful AUS visualization. Exploratory comparisons between visualization and non‐visualization groups were performed using the Mann–Whitney U test or Fisher's exact test. All analyses were considered exploratory, and no adjustment was made for multiple comparisons. All statistical analyses were performed using SPSS version 24 (IBM, Armonk, NY, USA) and EZR version 1.33 (Saitama Medical University, Jichi Medical University, Japan) [16].

## 3 Results

### 3.1 Patient Characteristics

**FIGURE 2:**
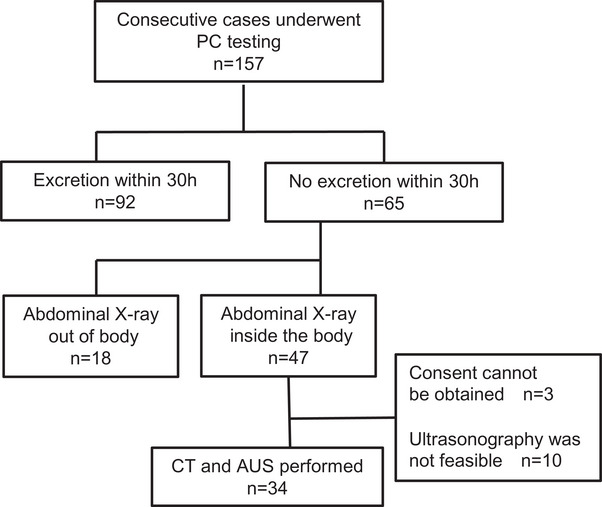
Study flow diagram. AUS, abdominal ultrasonography; CT, computed tomography; PC, patency capsule.

Of the 157 patients who underwent PC testing, PC excretion was confirmed in 110 cases. In three patients, informed consent was not obtained, and in 10 patients, AUS could not be performed because of the absence of an ultrasound technician or the unavailability of ultrasound equipment. Consequently, a total of 34 patients in whom PC excretion could not be confirmed were included in the final analysis. All 34 patients underwent both AUS and abdominal CT on the same day (Figure 2). The median interval between AUS and CT was 17.5 min (interquartile range, 16–22 min).

The study population consisted of 34 patients (15 men and 19 women), and detailed baseline characteristics are summarized in Table 1. The most common indication for capsule endoscopy was evaluation or follow‐up of Crohn's disease (*n* = 17), followed by investigation of anemia and suspected small‐bowel tumors.

**TABLE 1:** Clinical characteristics of the patients who underwent abdominal ultrasonography (AUS).

Number of patients	34
**Age (years), median (range)**	49 (10–84)
	(IQR 33.5–75.8)
**Sex, *n* (%)**	
Male	15 (44.1%)
Female	19 (55.9%)
**Indications for SBCE**	
Crohn's disease, established	12
Crohn's disease, suspected	5
Anemia	7
Small bowel tumor	4
SSBE	3
Ileus^a^	2
Abdominal pain	1

^a^ Ileus was not present at the time of examination.

Abbreviation: AUS, abdominal ultrasonography; SBCE, small‐bowel capsule endoscopy; SSBE, suspected small bowel bleeding.

### 3.2 Detection of Small‐bowel PC Retention

**FIGURE 3:**
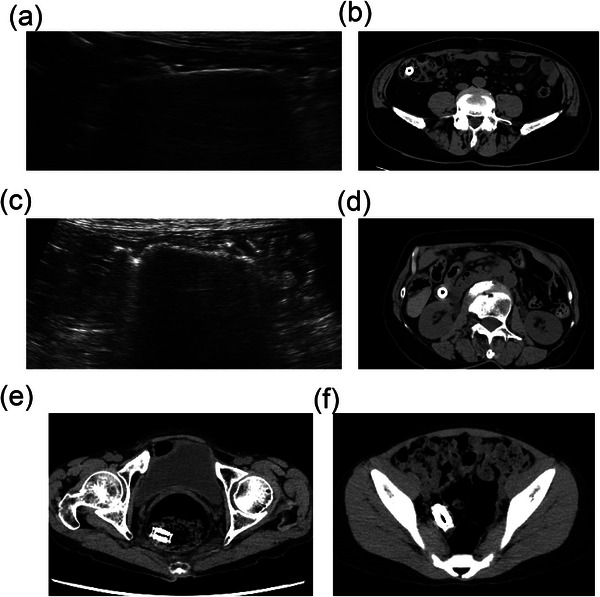
PC location identification by ultrasound and CT images. (a, b). Images of PC in the large intestine. PC was identified in the ascending colon, extending from the ileocecal junction. (c, d). Images of PC in the small intestine. PC was identified in the small intestine, where Kerckring folds were observed. (e) A case of PC buried in stool in the rectum, making it difficult to identify by ultrasound. (f) A case of PC located deep in the dorsal portion of the sigmoid colon, making it difficult to identify by ultrasound. PC, patency capsule; CT, computed tomography.

PC identification on AUS was based on the following criteria: visualization of a linear, high‐echoic structure measuring ≥20 mm, reproducible visualization during repeated scanning, and absence of deformation with probe compression (Figure 1b,c). Representative examples of PC localization by AUS and corresponding CT findings are shown in Figure 3a–d, and “not visualized” in Figure 3e,f.

AUS successfully visualized the PC in 28 of the 34 patients, with a visualization rate of 82.4%. The mean time required for PC identification was 7.6 min (range, 2–15 min). CT identified six patients with small‐bowel PC retention, all of whom were correctly classified as “small bowel**”** on AUS, resulting in a sensitivity of 100% (6/6; 95% CI, 61.0–100). No small‐bowel PC was misclassified or categorized as “not visualized.**”** All six patients with confirmed small‐bowel PC retention were judged to have a lack of functional gastrointestinal patency, and SBCE was not performed. All underwent double‐balloon endoscopy (DBE) for further evaluation, which identified an underlying stricture in five of the six patients (Crohn's disease–related stricture, *n* = 4; small‐bowel cancer, *n* = 1).

### 3.3 Small Bowel Versus Colon Classification

As shown in Table 2, among the 28 patients in whom the PC was located in the colon on CT, AUS classified 22 patients as “colon,” whereas the remaining six were categorized as “not visualized.” Using the predefined binary classification, in which AUS findings of “small bowel” were considered positive and findings of “colon” or “not visualized” were considered negative, AUS achieved a sensitivity of 100% (6/6; 95% CI, 61.0–100) for detecting small‐bowel PC retention and a specificity of 100% for identifying colonic PC location. The positive predictive value was 100% for both the small‐bowel and colon classifications.

**TABLE 2:** Diagnostic accuracy of abdominal ultrasonography (AUS).

Metric	Value	95% CI
Sensitivity (small‐bowel retention)	100% (6/6)	61.0–100
Specificity (CT‐negative for small‐bowel retention)	100% (28/28)	88.1–100
PPV (small bowel)	100% (6/6)	61.0–100
PPV (colon)	100% (22/22)	84.6–100
NPV	100% (28/28)	87.7–100
Accuracy	100% (34/34)	89.9–100

*Note*: For the primary endpoint (binary) analysis, AUS findings classified as “small bowel” were considered test‐positive. Test‐negative was defined operationally as failure to identify the PC within the small bowel on AUS (i.e., classified as “colon” or “not visualized”) and does not exclude small‐bowel retention.

Abbreviation: AUS, abdominal ultrasonography; PC, patency capsule; PPV, positive predictive value; NPV, negative predictive value.

### 3.4 Three‐Category Diagnostic Performance

In the category‐specific analysis comprising “small bowel,” “colon,” and “not visualized” classifications (Table 3), the positive predictive value was 100% for both the small‐bowel and colon categories. Sensitivity was 100% for the small‐bowel category and 78.6% for the colon category. All six cases classified as “not visualized” on AUS were confirmed to be located in the colon on CT.

**TABLE 3:** Category‐specific diagnostic performance.

AUS category	CT small bowel	CT colon	PPV (%)	95% CI (PPV)
Small bowel	6	0	100	61.0–100
Colon	0	22	100	84.6–100
Not visualized	0	6	—	—

AUS category	Sensitivity for CT‐confirmed location (%)	95% CI (Sensitivity)
Small bowel	100 (6/6)	61.0–100
Colon	78.6 (22/28)	60.5–89.9

Abbreviation: AUS, abdominal ultrasonography; CT, computed tomography; PPV, positive predictive value.

### 3.5 Six‐Segment Agreement Between AUS and CT

When PC location was evaluated across six anatomical segments, overall concordance between AUS and CT was 82.4% (28/34) (Table 4). Among the 28 patients in whom AUS successfully visualized the PC, segment‐level agreement with CT was 100%. For CT‐confirmed colonic PCs, AUS correctly identified the corresponding colonic segment in 22 of 28 patients, yielding a segment‐level identification rate of 78.6%.

**TABLE 4:** Six‐segment agreement between abdominal ultrasonography (AUS) and computed tomography (CT).

Segment (CT reference)	*n*	Correctly identified by AUS	Agreement (%)
Small bowel	6	6	100
Ascending colon	7	7	100
Transverse colon	4	4	100
Descending colon	8	8	100
Sigmoid colon	2	2	100
Rectum	1	1	100
Total (visualized cases)	28	28	100
Overall	34	28	82.4%

### 3.6 Body Mass Index and Age in Relation to PC Visualization

**FIGURE 4:**
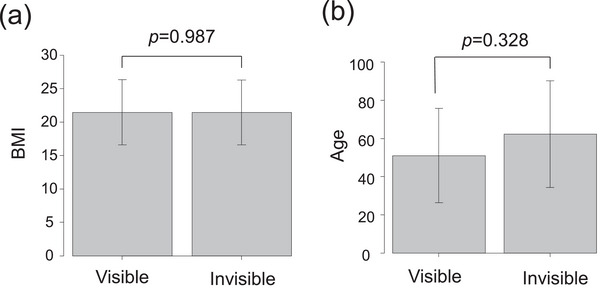
The effects of BMI and age on ultrasound visibility. (a) Ultrasound visibility and BMI. (b) Ultrasound visibility and age. BMI, body mass index.

Body mass index (BMI), age, and bladder urine volume have been reported as factors influencing ultrasonographic image quality [17]. In this study, BMI and age were compared between patients in whom the PC was successfully visualized and those in whom visualization was unsuccessful. Median BMI was 20.38 kg/m^2^ (interquartile range [IQR] 18.80–22.78) in the visualized group (*n* = 28) and 20.22 kg/m^2^ (IQR 17.75–24.06) in the non‐visualized group (*n* = 6) (*p* = 0.987) (Figure 4). Median age was 54 years (IQR 31.5–73) and 76 years (IQR 43–81), respectively (*p* = 0.328). No urinary retention was observed because all examinations were performed after voiding.

### 3.7 SBCE Outcomes

All patients judged by AUS to have colonic PC passage subsequently underwent SBCE, and no cases of capsule retention were observed.

## 4 Discussion

This prospective exploratory study showed that AUS demonstrated favorable performance in detecting small‐bowel PC retention, using CT as the reference standard. All patients with small‐bowel PC retention on CT were correctly identified by AUS, yielding a sensitivity of 100% (6/6; 95% CI 61.0%–100%). This finding indicates that AUS reliably detects patency failure, which represents the most clinically important outcome in pre–SBCE evaluation.

AUS findings directly influenced subsequent management. Patients with small‐bowel PC retention underwent DBE instead of SBCE, and clinically relevant strictures were identified in most cases. From a clinical pathway perspective, small‐bowel PC indicates insufficient patency and generally precludes SBCE, whereas colonic PC may allow SBCE without additional imaging. Non‐visualized findings should be considered indeterminate, with CT reserved for such cases or when clinical suspicion remains high. AUS visualized the PC in 82.4% of patients, suggesting that CT may be avoided in a substantial proportion of cases.

A notable strength of this study is the systematic assessment of AUS performance across both binary classification and six‐segment analysis. AUS successfully visualized the PC in 82.4%, and when visualization was achieved, segment‐level agreement with CT reached 100%. These results suggest that AUS not only identifies whether the PC has passed into the colon but can also accurately determine the specific colonic segment. Such detailed localization may support clinical decision making, particularly in cases with suspected partial obstruction through the gastrointestinal tract [18, 19, 20].

Non‐visualized findings require careful interpretation. All six non‐visualized cases (17.6%) were confirmed as colonic passage on CT. Non‐visualized findings should be interpreted as indeterminate and do not exclude small‐bowel retention; additional imaging, such as CT, may be warranted when clinical suspicion remains high. Most non‐visualized PCs were located in the transverse or sigmoid colon, reflecting the known technical limitation of AUS [10]. No patient judged by AUS to have colonic PC location experienced SBCE retention; however, further studies are needed to clarify the clinical implications of non‐visualized findings.

Previous studies have suggested the feasibility of identifying PCs by ultrasonography; however, most have been retrospective, lacked standardized reference imaging, or did not formally evaluate diagnostic accuracy metrics. Our study adds prospective evidence with rigorous comparison to CT, reinforcing AUS as a valid, radiation‐free alternative for PC assessment. Given the need to minimize radiation exposure, AUS may serve as a practical first‐line tool, reserving CT for inconclusive or high‐risk cases.

This study has several limitations. The apparently perfect accuracy observed in this study should be interpreted cautiously, given the small sample size and the limited number of small‐bowel retention cases. First, only six cases of CT‐confirmed small‐bowel PC retention were observed, resulting in a wide confidence interval for sensitivity and limiting the precision of the estimate. Second, this was a single‐center study, and the results may reflect operator expertise with gastrointestinal ultrasonography [21, 22]. Reproducibility among less experienced clinicians remains uncertain. Because AUS operators had access to prior radiographic information, incorporation bias cannot be completely excluded. In addition, AUS could not be performed in some eligible patients because of limited operator and equipment availability, which may limit generalizability. Future studies should assess interobserver variability, include operators with varying experience, and evaluate standardized training and imaging protocols. Third, visualization success was influenced by body habitus and bowel gas, factors that may vary across populations. Finally, the study population consisted only of patients who did not confirm PC excretion, which may limit applicability to all patients undergoing PC testing.

In conclusion, AUS appears to be a reliable and clinically useful modality for evaluating PC location before SBCE, with CT reserved for inconclusive or high‐risk cases. Further multicenter studies are warranted, particularly to define the optimal management of indeterminate (non‐visualized) findings.

## Author Contributions

**Tomoharu Matsuura**, **Satoshi Osawa**, and **Moriya Iwaizumi** contributed to the study's conception and design. **Wataru Inui**, **Tomoyuki Niwa**, **Kennichi Takahashi**, **Takatoshi Egami**, **Keisuke Inagaki**, **Tomohiro Takebe**, **Tatsuhiro Ito**, **Satoru Takahashi**, **Shunya Onoue**, **Yusuke Asai**, **Kiichi Sugiura**, and **Natsuki Ishida** contributed to performing the PC testing and thereafter SBCE. Material preparation, data collection, and analysis were performed by **Tomoharu Matsuura** and **Satoshi Osawa**. **Takanori Yamada** and **Yasushi Hamaya** contributed to the analysis and interpretation of data. **Ken Sugimoto** was involved in study supervision. **Tomoharu Matsuura** drafted the initial manuscript, and all authors revised it critically for important intellectual content. All authors approved the final manuscript version prior to submission.

## Funding

The authors have nothing to report.

## Ethics Statement

The Ethics Committee of Hamamatsu University School of Medicine in Japan reviewed and approved the study protocol (institutional review board approval number: 21‐245).

## Consent

Written informed consent was obtained from all participants.

## Conflicts of Interest

The authors declare no conflicts of interest.
